# Practice and Perception of First Aid Among Lay First Responders in a Southern District of India

**DOI:** 10.5812/atr.7972

**Published:** 2013-02-01

**Authors:** Uthkarsh Pallavisarji, Gopalkrishna Gururaj, Rao Nagaraja Girish

**Affiliations:** 1 Department of Community Medicine, Sri Siddhartha Medical College, Tumkur, Karnataka, India; 2 Department of Epidemiology, WHO Collaborating Centre for Injury Prevention and Safety Promotion, National Institute of Mental Health and Neuro Sciences, Bangalore, India, India; 3 Department of Epidemiology, National Institute of Mental Health and Neuro Sciences, Bangalore, India

**Keywords:** Emergencies, First-Aid, First Responders, Injuries, Prehospital Care

## Abstract

**Background:**

Injuries rank among the leading causes of morbidity and mortality worldwide, and are steadily increasing in developing countries like India. However, it is often possible to minimize injury and crash consequences by providing effective pre-hospital services promptly. In most low-and middle-income countries (LMICs), transportation of road traffic victims, is usually provided by relatives, taxi drivers, truck drivers, police officers and other motorists who are often untrained.

**Objectives:**

The current study was conducted to understand the current practice and perception of first aid among lay first responders in a rural southern district of India.

**Materials and Methods:**

The current cross sectional descriptive study was conducted in the southern district of Tumkur in India within three months from January to March 2011 and covered the population including all police, ambulance personnel, taxi drivers, bus and auto drivers, and primary and middle school teachers within the study area.

**Results:**

Nearly 60% of the responders had witnessed more than two emergencies in the previous six months and 55% had actively participated in helping the injured person. The nature of the help was mainly by calling for an ambulance (41.5%), transporting the injured (19.7%) and consoling the victim (14.9%). Majority (78.1%) of the responders informed that they had run to the victim (42.4%) or had called for an ambulance. The predominant reason for not providing help was often the ‘fear of legal complications’ (30%) that would follow later. Significant number (81.4%) of respondents reported that they did not have adequate skills to manage an emergency and were willing to acquire knowledge and skills in first aid to help victims.

**Conclusions:**

Regular and periodical community-based first aid training programs for first care responders will help to provide care and improve outcomes for injured persons.

## 1. Background

Injury, an increasingly significant public health issue worldwide, accounts for up to 16% of the global burden of disease, with road traffic injuries (RTIs), in particular, on the rise (1). By 2030, road traffic crashes are predicted to be the eighth-leading cause of death and fourth-leading cause of disability-adjusted life years worldwide (2, 3). Currently, more than 90% of road traffic injury deaths occur in Low and Middle Income Countries (LMICs) (4, 5). According to WHO, RTIs are the sixth leading cause of death in India with a greater share of hospitalizations, deaths, disabilities and socioeconomic losses in young and middle-age populations (6) where approximately 50% of injury deaths occur in the prehospital setting (7). Prevention and control of RTIs require multisectoral integrated actions aiming at limiting the occurrence of crashes, providing best possible care in the event of a crash (before reaching the hospital and in the health care facility) and suitable rehabilitation services for the injured person (8). However, it is often possible to minimize crash consequences by providing effective pre-hospital services promptly (9). Each year, among the 1.2 million lives lost globally, many lives could be saved and many of the ensuing disabilities suffered by the 50 million injured could be prevented, if rapid and competent pre-hospital services were available at the crash scene (10). Prehospital services are a continuum of activities at the crash site and till the injured person is adequately managed by hospital staff. First care responders, the ones who are first at the crash site, can take necessary steps for safety and smooth management, especially where transportation systems are yet to develop. In most low-and middle-income countries (LMICs) like India, transportation of road traffic victims, is usually provided by relatives, drivers of private vehicle (three wheeled autorickshaws, taxis and other local vehicles), police officers, and other motorists who are usually untrained (11, 12). Ambulances, if available, usually exist only in urban areas (10) and it takes them a long time to arrive in rural areas. Studies have shown that the inadequacy of public health infrastructure and poor access to health services are important reasons for the high burden of RTIs and/or their severity (13). One of the most common observations in relation to Pre-hospital care is the interaction of untrained lay people and their lack of knowledge and skills in handling the situation in general and the victims in particular.

## 2. Objectives

The current study aimed to understand the current practice and perception of first aid among such lay people and provide a basis for strengthening prehospital care system.

## 3. Materials and Methods

### 3.1. Study Area and Study Population Injuries

This study was done in Tumkur, which is one of the 29 districts of Karnataka state, in southern part of India situated to the North-west of Bengaluru at a distance of about 70 kms. With a population of 2.58 million, the district occupies 10598 sq km area, with a total road length of 13738 kms (196 kms National highways, 754 kms State highways, 3761 kms of Major district roads).Tumkur has the 9th largest district road network in the state and to the residents of nearly 15 districts in Karnataka is the gateway to Bangalore. The district also has a 400 bed district hospital, 4 community health centres, 139 primary health centres and a number of private hospitals and local practitioners. Emergency care is provided by nearly 25 ambulances belonging to the state government run (EMRI-Emergency Management and Research Institute) services. The subjects under study included 186 auto drivers (local three wheeler vehicles), 215 teachers (primary and middle school teachers), 167 bus drivers, 91 police and 61 ambulance personnel drawn from the entire district. All participating members were contacted individually and agreed to participate in the study. Verbal consent was obtained from all participants after explaining the purpose, objectives and nature of the study. The participants’ place of residence was spread over the total district, even though the district headquarters formed their place of work.

### 3.2. Study Design and Data Collection

A preliminary survey was done to collect relevant details on the number and locations of all the primary and middle schools along with the number of auto, bus, taxi and ambulance stations in the study area. Drivers of autos, taxis, and buses were contacted for the study at their respective stations, during their work break. Teachers of primary and middle school were contacted in their schools during the monthly meeting of teachers, and ambulance personnel were contacted at hospitals. A cross-sectional study was undertaken by interviewing selected participants on a one to one basis after obtaining informed consent. Data collection was done by a team of trained medical interns (trainees) proficient in local language. A semi structured pilot tested questionnaire was used to collect the data. Information on types and frequencies of emergencies witnessed and type of help provided was collected from all study subjects except ambulance personnel. Information on barriers to first aid provision, history of training in first aid and current availability of first-aid supplies and perception on core areas of scene safety, bleeding control, airway evaluation, recovery position for unconscious patients, and safe transportation was collected from all study participants.

### 3.3. Statistical Analysis

SPSS version 16 was employed to analyze the data.

## 4. Results 

A total of 720 subjects participated in the study and among them 76% were men. The study population included teachers (30%), drivers (auto drivers – 26%; bus drivers - 24%) and ambulance drivers (8%). Nearly 9 out of 10 survey participants were literates with completion of basic education (Table 1).

**Table 1.** Socio Demographic Characteristics of Participants

Socio Demographic Variables	No. (%)
**Gender**	
Male	547 (75.88)
Female	173 (24.12)
**Education**	
Illiterates	61 (8.42)
Literates	659 (91.58)
**Occupation**	
Auto drivers	186 (26)
Teachers	215 (30)
Bus drivers	167 (24)
Police	91 (12)
Ambulance personnel	61 (8%)

### 4.1. Emergencies Witnessed and Care Provided by the First Responders

**Figure 1.**
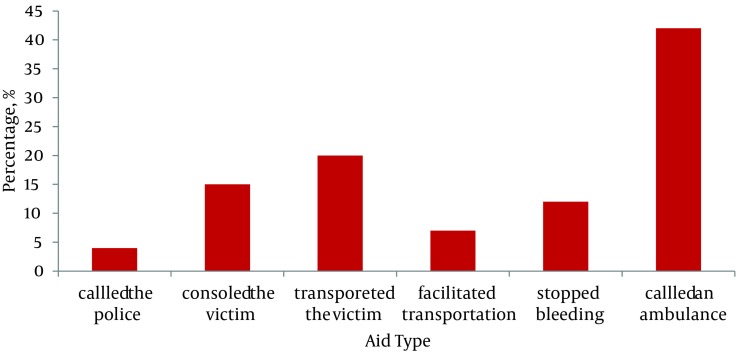
Common Aid Given by First Responders

Nearly 52 % (372) had witnessed more than two emergencies during the last six months. The most common emergencies witnessed were road traffic injuries (52%), others were burns, poisoning, cardiac emergencies, and pregnancy. Nearly 44.6% (317) had been called more than once to provide help in emergencies and more than half (33.5 - 47 %) reported providing some assistance. Most common aid provided was calling an ambulance (41.5%) and nearly 90% of the participants were aware of the locally available ambulance number, viz 108 belonging to the public ambulance systems (Figure 1). Ambulance (48.7%) and auto (40%) were commonly used to transfer cases to hospitals. Most commonly, cases were shifted to a nearby government hospital (50%) or private nursing homes (50%). Nearly 50% had reported that they took more than an hour to reach a hospital in the last emergency they handled.

### 4.2. Current Capacity of Participants

**Figure 2.**
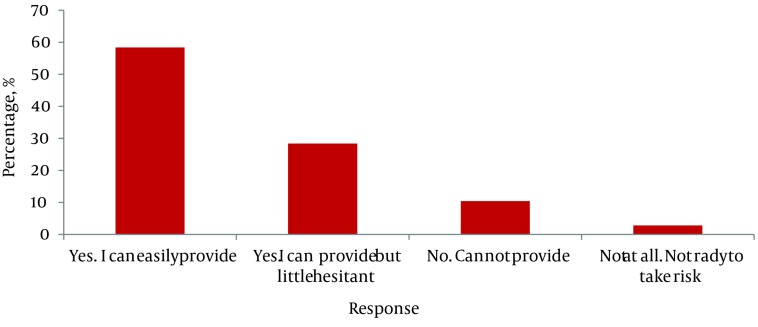
Confidence of First Responders to Provide First Aid

All ambulance personnel including nearly 26% of other study participants had undergone some type of first aid training earlier and only 13% had undergone training in the last two years. The majority (62%) of drivers had training before issuance of their licenses and 58% of them felt confident enough to provide aid without any hesitance (Figure 2).

### 4.3. Barriers to Provide First Aid

Nearly 37% of the participants had withheld themselves once and 35% more than once from rendering any help when called during an emergency in the past six months. Reasons were several and are provided in Table 2. Not knowing precisely what to do and fear of the scene was the common reason cited by 79 (30 %) of the respondents, followed closely by an equal number due to fear of medico legal reasons and nonavailability of any first aid supplies was also a reason as informed by 20 respondents (Table 2). Knowledge of participants in areas of scene safety, bleeding control, airway maintenance, recovery position for unconscious patients, and safe transportation was as depicted in Table 3. Rushing to the victim (42.5%) and calling 108 (emergency number) (42%) was perceived as the first step to be taken at the scene of emergency. Nearly 55% felt that assessing consciousness of the victim was the first thing in victim evaluation. Tying the cloth or bandage (76%) at the bleeding site and tied down on their back (68.5%) were perceived as the best way to control bleeding and to transport the unconscious respectively. Nearly 61% felt driving at a faster speed to a nearby hospital will help in better survival. Drivers had better knowledge in prehospital care and they were more willing to attend training programs on first aid compared to teachers and police (P < 0.0001).

**Table 2.** Reasons for not Providing First Aid

Reasons for not Providing Aid	Frequency, No. (%)
Did not know what to do	79 (29.8)
Scared of doing harm than help	52 (19.6)
No materials to provide first aid	20 (7.6)
Scared of legal complications	79 (29.8)
Other reasons	35 (13.2)
Total	265 (100)

**Table 3.** Perception of Prehospital Care

	Percentage, %
**What is the first step you should take at the scene**	
Run to the Victim	42.5
Call 108	42
Make sure the scene is safe for you and others	13.8
Drive Carefully past it	2
**When evaluating an injured patient, what is the first thing you should do?**	
Assess Whether patient is conscious or not	55
Check airway and breathing	25.5
Check for bleeding	5
Scoop and rush the victim to hospital	14.5
The best and safest way to stop bleeding is	
Tying the cloth or bandage at the bleeding site	75.8
Pour water on wound	10.8
Direct pressure and elevation of limb	4.2
Elevation and tying the cloth or bandage at the bleeding site	9.2
**The best position for transporting an unconscious patient is**	
Tied down on their back	68.5
On their side	11.9
On their abdomen	5.2
Sitting upright	14.4
**Driving at faster speeds to a health facility results in better survival of patient**	
Yes	61
No	38.8

## 5. Discussion

It is well acknowledged that emergency care systems are generally poor in LMICs (14) and more of an urban phenomenon in countries like India (15). Pre-hospital care is the care provided in the community and at the crash site (at home, school, work, or recreation area) or even during transportation until the patient arrives at a formal health-care facility capable of providing definitive care. One of the most common issues raised in relation to prehospital care is the interaction and participation of untrained local lay people and their lack of knowledge and skills in handling the situation in general and the victims in particular. According to the World Health Organization (WHO) (16), the role of lay people who are present at a crash scene should be: to contact the emergency services; help to put out fires; and take action to secure the crash scene (e.g. preventing further crashes, preventing harm to rescuers and bystanders, controlling the crowd of onlookers, and applying first aid). This study was done to know the current practice and perception of first aid in such lay people. Nearly 60% of participants other than ambulance personnel had witnessed more than two emergencies in last six months and 55% had provided some sort of help in contrast to a study in Kampala where 90% had provided some assistance (16), adding to the fact that lay people regularly witness emergencies and they can provide help in prehospital settings. In the current study, it was observed that the most common aid provided was calling an ambulance (42%) in contrast to study in Kampala where lifting/moving (82%) the victims was common (16). In the current study, most commonly lay first responders transported cases to a nearby government hospital (50%) or private nursing homes (50%) which most often lacks the definitive care, leading to delay in definitive care (17, 18). Other than ambulance autos (40%) and other private vehicles were commonly used to transfer cases to hospitals which is similar to other studies (19-21) showing that auto drivers are the first responders in most of the emergencies where ambulance personnel are not available. Taking an injured person to a hospital is considered the most effective way to save lives (22) and in the present study, nearly 50% have reported that it took them more than an hour to reach a hospital in the last emergency they handled, which is consistent with the other studies (17-19). Delays in availability of definitive care could be an important reason for poor and negative outcomes in many LMICs. Nearly 58% of study participants felt confident enough to provide first aid, nearly 45% of those who were confident to provide aid had some experience of first aid training. Other than ambulance personnel who were trained during their professional course, training of other first responders in first aid was merely a brief orientation to first aid without much focus on how to handle an emergency. This type of training does not provide the real knowledge and skills required to handle emergencies confidently. Most of the participants including few ambulance personnel reported that the most common reason for not providing aid was, lack of knowledge (15, 21) and fear of legal complications which emphasizes the need for more focused training programs to increase their knowledge and skills in providing first aid. Though ambulance personnel were trained the lack of confidence among them emphasizes the need for periodic remedial instructions to update them in the field of first aid. Significant numbers of neurological injuries appear to be a result of the extrication process or victim transportation without adequate immobilization (13, 23, 24), generally by untrained people (25). In the current study, participants (68%) had knowledge on correct position of victim transportation but many did not know the correct method of bleeding control. In the current study, only half of the participants were aware of scene safety and nearly 90% were aware of common emergency number (n = 108). Several studies in LMICs especially in settings with a high burden of injuries, have demonstrated the effectiveness of training lay people in first aid (12, 22). In the current study, though the majority of participants had witnessed emergencies more than once and nearly half of them been called to help during emergencies, only 34% had undergone some sort of training which is less compared to the study in kampala (16) and only 13% had training in the last two years. Nearly 42% of first responders were not confident enough to provide any sort of first aid, stressing the importance of regular training programs for these first responders. Limitations of the preliminary study include selection and interview bias that might have contributed to study results. More in-depth studies that focus on skills assessment among different categories of personnel are required to develop scientific and culturally appropriate lay first responder training programs along with strengthening other components of prehopsital care. There is no formal prehospital care system in the area under study. Lay people commonly witness emergencies and call for help, they are not having any formal training in first aid. Lack of knowledge in first aid and fear of legal complications is preventing them from providing first aid. A formal compulsory regular training of lay first aid providers may improve the practice of first aid.

## References

[1] Lopez AD, Mathers CD, Ezzati M, Jamison DT, Murray CJ (2006). Global and regional burden of disease and risk factors, 2001: systematic analysis of population health data.. Lancet..

[2] Mathers CD, Loncar D (2006). Projections of global mortality and burden of disease from 2002 to 2030.. PLoS Med..

[3] WHO. (2004). The global burden of disease: 2004 update..

[4] Hofman K, Primack A, Keusch G, Hrynkow S (2005). Addressing the growing burden of trauma and injury in low- and middle-income countries.. Am J Public Health..

[5] Murray CJL, Lopez AD (1996). Global health statistics: a compendium of incidence prevalence and mortality estimates for over 200 conditions..

[6] Ministry of Health and Family Welfare. (2004). Integrated Disease Surveillance Project: Project Implementation Plan 2004–09..

[7] Gururaj G (2008). Road traffic deaths, injuries and disabilities in India: current scenario.. Natl Med J India..

[8] Peden M, Scurfield R, Sleet D, Mohan D, Hyder AA, Jarawan E (2004). World Report on Road Traffic Injury Prevention..

[9] Elvik R, Hoye A, Vaa T, Sorensen M (2009). Handbook of road safety measures Amsterdam..

[10] von Elm E (2004). Prehospital emergency care and the global road safety crisis.. JAMA..

[11] Kobusingye OC, Hyder AA, Bishai D, Hicks ER, Mock C, Joshipura M (2005). Emergency medical systems in low- and middle-income countries: recommendations for action.. Bull World Health Organ..

[12] Mock CN, Tiska M, Adu-Ampofo M, Boakye G (2002). Improvements in prehospital trauma care in an African country with no formal emergency medical services.. J Trauma..

[13] Mohan D, Tiwari G, Meleckidzedeck K, Fredrick MN (2006). Road traffic injury prevention training manual Geneva..

[14] (1989). UK medical research handbagged again.. Lancet..

[15] Joshipura M, Hyder AA, Rehmani R (2004). Emergency care in South Asia: challenges and opportunities.. J Coll Physicians Surg Pak..

[16] Jayaraman S, Mabweijano JR, Lipnick MS, Caldwell N, Miyamoto J, Wangoda R (2009). Current patterns of prehospital trauma care in Kampala, Uganda and the feasibility of a lay-first-responder training program.. World J Surg..

[17] Macintyre K, Hotchkiss DR (1999). Referral revisited: community financing schemes and emergency transport in rural Africa.. Soc Sci Med..

[18] Carr BG, Caplan JM, Pryor JP, Branas CC (2006). A meta-analysis of prehospital care times for trauma.. Prehosp Emerg Care..

[19] Uthkarsh PS, Suryanarayana S, Gautham S, Murthy N, Pruthvish S (2012). Status of pre-hospital care among injury cases admitted to a Tertiary hospital in South India.. Int J Crit Illn Inj Sci..

[20] Gururaj G (2008). National Institute of Mental Health and Neur Bengaluruo Sciences..

[21] Shaw B, Menon GR, Gururaj G (2009). evelopment of feasibility module for road traffic injuries surveillance.. ICMR Bulletin..

[22] Husum H, Gilbert M, Wisborg T, Van Heng Y, Murad M (2003). Rural prehospital trauma systems improve trauma outcome in low-income countries: a prospective study from North Iraq and Cambodia.. J Trauma..

[23] Podolsky S, Baraff LJ, Simon RR, Hoffman JR, Larmon B, Ablon W (1983). Efficacy of cervical spine immobilization methods.. J Trauma..

[24] Cloward RB (1980). Acute cervical spine injuries.. Clin Symp..

[25] Wilmink AB, Samra GS, Watson LM, Wilson AW (1996). Vehicle entrapment rescue and pre-hospital trauma care.. Injury..

